# Bixin Attenuates Experimental Autoimmune Encephalomyelitis by Suppressing TXNIP/NLRP3 Inflammasome Activity and Activating NRF2 Signaling

**DOI:** 10.3389/fimmu.2020.593368

**Published:** 2020-12-09

**Authors:** Ye Yu, Dong-Ming Wu, Jing Li, Shi-Hua Deng, Teng Liu, Ting Zhang, Miao He, Yang-Yang Zhao, Ying Xu

**Affiliations:** Clinical Medical College, The First Affiliated Hospital, Collaborative Innovation Center of Sichuan for Elderly Care and Health of Chengdu Medical College, Chengdu, China

**Keywords:** bixin, experimental autoimmune encephalomyelitis, thioredoxin-interacting protein, NLRP3, nuclear factor erythroid 2-related factor 2, reactive oxygen species

## Abstract

Multiple sclerosis (MS), an autoimmune and degenerative disease, is characterized by demyelination and chronic neuroinflammation. Bixin is a carotenoid isolated from the seeds of *Bixa orellana* that exhibits various potent pharmacological activities, including antioxidant, anti-inflammatory, and anti-tumor properties. However, the effects of bixin on MS have not yet been examined. To evaluate the effects and underlying molecular mechanisms of bixin on MS, experimental autoimmune encephalomyelitis (EAE) was established in C57BL/6 mice, which were treated *via* intragastric administration of bixin solutions. To evaluate the molecular mechanisms of bixin, quantitative reverse-transcription PCR, western blot, immunohistochemistry, flow cytometry, and enzyme-linked immunosorbent assay analyses were performed. We found that bixin significantly improved the symptoms and pathology in EAE mice, reduced the release of inflammatory cytokines TNF-α, IL-6, IL-8, IL-17, and IFN-γ, and increased the expression of the anti-inflammatory cytokine IL-10. And bixin reduced the proportion of Th1 and Th17 cells in the spleen and CNS, and suppressed microglia aggregation, and TXNIP/NLRP3 inflammasome activity by scavenging excessive reactive oxygen species (ROS) in EAE mice. Furthermore, bixin inhibited inflammation and oxidative stress *via* activating nuclear factor erythroid 2-related factor 2 (NRF2), and its downstream genes in EAE mice, meanwhile, these effects were suppressed upon treatment with an NRF2 inhibitor, ML385. Bixin prevented neuroinflammation and demyelination in EAE mice primarily by scavenging ROS through activation of the NRF2 signaling pathway. Taken together, our results indicate that bixin is a promising therapeutic candidate for treatment of MS.

## Introduction

Multiple sclerosis (MS) is a chronic autoimmune and degenerative disease of the central nervous system (CNS), and the most frequent cause of neurological disability in young adults (1). The pathological changes in patients with MS are multifaceted, including multiple demyelinating plaques, and are accompanied by glial activation and axonal damage (2–6). Although many studies have proposed possible molecular mechanisms and therapeutic strategies for MS, its pathology is largely unknown, involving apoptosis, oxidative stress, and inflammation. As such, effective treatments remain elusive (7, 8).

A recent study has proven that the NLRP3 inflammasome plays a pivotal role in the pathogenesis of neuroinflammation and demyelination in EAE (5). The thioredoxin-interacting protein (TXNIP)-NLRP3 inflammasome is a macromolecular polyprotein complex, composed of TXNIP, NLRP3, the junction protein ASC, as well as the effector protein caspase-1. Excessive reactive oxygen species (ROS) accumulation, induced by high glucose levels, increases TXNIP expression. Accumulated TXNIP, in turn, activates the NLRP3 inflammasome, consequently inducing the secretion of IL-1β and IL-18 as part of the inflammatory response (9–12). Furthermore, activated NLRP3 inflammasomes induce the expression of gasdermin D (GSDMD), which leads to the release of numerous inflammatory cytokines, exacerbating the detrimental inflammatory response (13). Hence, blocking TXNIP-NLRP3 activity is expected to alleviate neuroinflammation and demyelination.

Bixin is a natural chemo-preventive carotenoid isolated from the seeds of *Bixa orellana* that is capable of crossing the blood-brain barrier (14–16). Bixin is a lineal apocarotenoid of 25 carbon atoms with nine double bonds and has demonstrated various potent pharmacological activities, including antioxidant, anti-inflammatory, and anti-tumor properties (17, 18). Previous studies have demonstrated that bixin can ameliorate high-fat-diet-induced cardiac injury and PM2.5 particle-induced lung injury through suppressing inflammation and oxidative stress (19, 20). The present study evaluated the effects of bixin on neuroinflammation and demyelination in a mouse model of autoimmune encephalomyelitis, with the aim of assessing whether bixin could serve as an effective therapeutic compound for MS.

## Materials and Methods

### Antibodies

The primary antibodies used for western blot assay and immunohistochemical analysis were as follows: anti-GAPDH (10494-1-AP), anti-CD3 (17617-1-AP), anti-MBP (10458-1-AP), anti-IBA1 (10904-1-AP), anti-CD68 (28058-1-AP), and horseradish peroxidase (HRP)-conjugated secondary antibodies (SA00001-2) used for western blot analysis were purchased from Proteintech (Wuhan, China); anti-Catalase (CAT, 14097), anti-SOD2 (13141S) (Cell Signaling Technology, Danvers, MA, USA); anti-3-Nitrotyrosine (3-NT, AB5411, Millipore); anti-NAD(P)H dehydrogenase quinone 1 (NQO-1, ab34173), anti-NRF2 (ab31163), anti-TXNIP (ab232330), anti-NLRP3 (ab4207), anti-Caspase-1 (ab179515), anti-IL-18 (ab68435), anti-IL-1β (ab2105), and anti-ASC (ab127537) were purchased from Abcam (Cambridge, UK);. Cy3 goat anti-rabbit IgG (H+L) (A0516) and Alexa Fluor 488 goat anti-mouse IgG (H+L) (A0428) used for immunofluorescence analysis were purchased from Beyotime (Shanghai, China). The SPlink detection kits used for immunohistochemical analysis were purchased from ZSGB-BIO Technology (SP-9000) (Beijing, China). The antibodies used for flow cytometry analysis were as follows: PE-CYANINE anti-mouse IL-17 (25–7177–80), PE-CYANINE anti-mouse IFN-γ (25-7311-82), FITC anti-mouse CD4 (11-0041-82) (eBioscience, San Diego, CA, USA).

### Establishment of the EAE Model

Seven-week-old female C57BL/6 mice were purchased from Chengdu Dashuo Experimental Animal Company (Chengdu, China). All animal procedures were approved by the Animal Policy and Welfare Committee of Chengdu Medical College (CDYXY-2019036). All mice were housed in a specific pathogen-free facility with a 12-h light/dark cycle and provided with regular food and water during the acclimatization period.

The EAE model was established as previously described (21). Myelin oligodendrocyte glycoprotein_35–55_ (MOG_35–55_) peptide (Hook Labs, USA) (200 μg) was dissolved in 100 μl of phosphate-buffered saline (PBS) and emulsified with 100 μl of complete Freund’s adjuvant (CFA; Chondrex, USA) supplemented with 400 μg *Mycobacterium tuberculosis* H37Ra (Difco, BD Biosciences, USA). Next, the above emulsions were subcutaneously injected into the mice (Day 1). Pertussis toxin (PTX; List Biological Labs, Campbell, CA, USA) (300 ng) was intraperitoneally administered on the first and third days post-immunization.

### Bixin Treatment

Animals were randomly divided into the following five groups: healthy control (PBS, n = 5), EAE (n = 5), EAE + bixin (50 mg/kg, once daily; n = 5), EAE + bixin (100 mg/kg, once daily; n = 5), and EAE + bixin (200 mg/kg, once daily; n = 5). Bixin (6983-79-5, MedChemExpress, Shanghai, China) was dissolved in dimethyl sulfoxide (DMSO) at 2 g/ml and then diluted with PBS. Starting at 12 days post-immunization, mice in the EAE + bixin group were administered intragastrically with bixin solutions (50, 100, or 200 mg/kg/day) for 18 days. On day 30 post-immunization, the mice from each group were euthanized, the spinal cords, brains, spleens, and peripheral blood were collected and used for further experiments.

### NRF2 Inhibitor Treatment

The NRF2 inhibitor ML385 (MCE, Shanghai, China) was dissolved in DMSO at 300 mg/ml and diluted with PBS (22). Mice were randomly assigned to the following eight groups: healthy control (PBS, n = 5), EAE (n = 5), EAE + bixin (100 mg/kg, once daily; n = 5), bixin (100 mg/kg, once daily; n = 5), ML385 (n = 5), ML385 + EAE (n = 5), ML385 + EAE + bixin (100 mg/kg, once daily; n = 5), and ML385 + bixin (100 mg/kg, once daily; n = 5). ML385 (30 mg/kg) pre-treatment was intraperitoneally administered 1 h before intragastric administration of bixin.

### Bodyweight and Behavioral Assessments

Clinical behavior scores of each group were evaluated as per the following criteria: 0, without symptoms; 1, loss of tail tension; 2, flaccid hind limb; 3, moderate hind limb paralysis; 4, paralysis of both hind limbs and forelimbs, or accompanied with urinary and fecal disorders; and 5, near-death state (2). The body weight was recorded daily.

### Hematoxylin and Eosin (H&E) and Luxury Fast Blue (LFB) Staining

Mice were anesthetized using 0.6% pentobarbital sodium (40 mg/kg), and the lumbar enlargements of spinal cords and the right hemispheres of brains tissues were collected and fixed with 4% paraformaldehyde (in PBS) for 24 h at room temperature, dehydrated with an ethanol gradient and cleared with xylene, subsequently embedded in paraffin, and then cut into 5-μm sections.

To evaluate the degree of inflammatory cell infiltration, brain sections and spinal cords were stained using an H&E staining kit (Beyotime Biotechnology, Shanghai, China). The sections were dewaxed and dehydrated, subsequently washed with PBS, and then stained with hematoxylin and eosin for 2 min, respectively. The inflammatory infiltration was evaluated as previously reported (23), 0 = no inflammatory cells; 1 = a few scattered inflammatory cells; 2 = organization of inflammatory infiltrates around blood vessels; 3 = extensive perivascular cuffing with extension into parenchyma.

The spinal cords were stained with LFB staining solution (Solarbio, Beijing, China), to evaluate changes in demyelination. The sections were stained with modified page staining solution and page peach red dye solution, respectively, after being dewaxed and dehydrated with an ethanol gradient. The demyelination scores were evaluated as previously reported (23), 0 = none; 1 = rare foci; 2 = a few areas of demyelination; and 3 = large areas of demyelination. The images were randomly captured at 20× magnification (XI 71 Olympus, Tokyo, Japan).

### Bielschowsky Staining

To determine the degree of axon degeneration, the spinal cords sections were stained using a Bielschowsky staining kit (Bioss, Beijing, China). The sections were dewaxed and dehydrated, subsequently washed with distilled water, and then stained with Bielschowsky silver nitrate solution in a 37°C incubator for 30 min, Bielschowsky ammonia silver solution for 20 s, gold chloride solution for 3 min, respectively. The images were randomly captured at 20× magnification (XI 71 Olympus, Tokyo, Japan).

### Immunohistochemical Analysis (IHC)

IHC was performed using an SP link detection kit (ZSGB-BIO Technology, Beijing, China). Tissue sections were dewaxed and dehydrated and washed with PBS. Subsequently, the samples were boiled in a citrate buffer (pH 6.0) for antigen retrieval and blocked using 5% normal goat serum at 37°C for 1 h. The sections were then incubated at 4°C overnight with primary antibodies (1:200). After washing with PBS, the sections were then incubated with the corresponding secondary antibody for 30 min. Finally, diaminobenzidine was used as the chromogen to visualize the immunocomplexes, and then the sections were counterstained with hematoxylin. Images of the random brain and spinal cord sections were captured at 40× magnification and 20× magnification, respectively (XI 71 Olympus, Tokyo, Japan).

### Immunofluorescence Staining (IF)

The lumbar enlargements of spinal cords and the right hemispheres of brains tissues were embedded in OCT and cut into 10-μm sections for IFC staining. After washing with PBS, tissue sections were blocked with 5% BSA for 30 min at 37°C, and then incubated overnight at 4°C with primary antibodies. After washing, the sections were incubated with secondary antibodies Cy3 goat anti-rabbit IgG (H+L) or Alexa Fluor 488 goat anti-mouse IgG (H+L) (1:200) for 1 h at 37°C, and nuclei were stained with 4′,6-diamidino-2-phenylindole (DAPI). Fluorescent images were captured at 40× magnification with a fluorescence microscope.

### Quantitation of Oxidative Stress

Dihydroethidium (DHE; Molecular Probes, Eugene, OR, USA) staining was used to detect ROS levels in the lumbar enlargements of spinal cords and the right hemispheres of brains tissues. The sections were dewaxed and dehydrated with an ethanol gradient, after washing with PBS (pH 7.4), tissue sections were blocked using 5% BSA for 30 min at 37°C, and stained with 5 μmol/L DHE (in PBS) for 30 min at 37°C. The ROS levels in EAE mice, in the absence or presence of bixin, were likewise evaluated by DHE staining. Finally, fluorescence images of brain or spinal cord sections were randomly captured at 20× magnification (XI 71 Olympus, Tokyo, Japan), and the fluorescent intensity was analyzed by Image J software.

### Cytokine Quantification *via* ELISA

Peripheral blood was collected and centrifuged at 3,000 rpm for 20 min, a portion of the serum was used for quantitating IL-1β, IL-18, IL-17, and IFN-γ levels by ELISA, while the remaining serum maintained −80°C for further analysis. The corresponding ELISA kits were used to assay IL-1β, IL-18, IL-17, and IFN-γ levels (MIBIO Biotechnology, Shanghai, China).

### Measurement of Superoxide Dismutase (SOD), Malondialdehyde (MDA)

The remaining serum of each mouse was used to detect the activities of SOD and the levels of MDA (The Institute of Biological Engineering of Nanjing Jiancheng, Nanjing, China). The protocol was according to the manufacturer’s instructions.

### Flow Cytometry

The CNS tissues and spleens were collected and mononuclear cells (MNCs) were isolated as previously reported (23, 24). CNS tissues were digested in dulbecco’s modified eagle medium (DMEM) containing 2 mg/ml collagenase II (Sigma-Aldrich, St. Louis, MO, USA) and 1 mg/ml DNAseI (Sigma-Aldrich, St. Louis, MO, USA) at 37°C for 30 min; MNCs were prepared by passing the tissue through a 100 μm cell strainer and density gradient centrifugation using Percoll (30/70%)(Sigma-Aldrich, St. Louis, MO, USA); MNCs were collected from the interface, washed, and resuspended in culture medium for further analysis. Splenocytes were isolated from the spleens of mice after being homogenized and passed through a 100 μm cell strainer. To measure the percentages of Th1 and Th17 cells, MNCs were stimulated with 50 ng/ml PMA, 500 ng/ml ionomycin containing GolgiPlug™ (BD Biosciences, San Jose, CA, USA) for 4 h at 37°C, then permeabilized with Perm/Fix solution, next stained with IL-17 and IFN-γ antibody at 4°C for 30 min, respectively; finally, the cells were stained with CD4 antibody at 4°C for 30 min. Cells were analyzed by BD Accuri C6 Flow cytometry (BD Biosciences, San Jose, CA, USA) and the data was analyzed by BD Accuri C6 software (BD Biosciences, San Jose, CA, USA).

### Quantitative Reverse-Transcription PCR (qRT-PCR)

Total RNA was extracted from the spinal cords tissue using a total RNA extraction kit (Solarbio, Beijing, China) according to the manufacturer’s instructions. Next, cDNA was synthesized using an iScript cDNA synthesis kit (Bio-Rad, Hercules, CA, USA). *Nrf2*, *Cat*, *Nqo1*, *Txnip*, *Nlrp3*, *Asc*, *Casp1*, *Il18*, *Il1β*, *Il6*, *Il8*, *Il10*, *Tnfα*, *Il17*, *Ifnγ*, and *Sod2* mRNA levels were analyzed by qRT-PCR with SYBR Green Supermix (Bio-Rad, Hercules, CA, USA). The primers were synthesized by Shanghai Shenggong and are listed in **Table 1** (*Actb* was used as an internal control for quantitation). The 2^-ΔΔCT^ method was used to calculate relative mRNA levels.

**Table 1:** Primer sequence information.

Gene	Forward primer (5’→3’)	Reverse primer (5’→3’)
*Actb*	GTGCTATGTTGCTCTAGACTTCG	ATGCCACAGGATTCCATACC
*Il6*	CTTGGGACTGATGCTGGTGACAAC	AGGTCTGTTGGGAGTGGTATCCTC
*Il8*	CATGGGTGAAGGCTACTGTTGGC	GCTTCATTGCCGGTGGAAATTCC
*Tnfα*	TCTACTGAACTTCGGGGTGATCGG	GTGGTTTGTGAGTGTGAGGGTCTG
*Il10*	CACTGCTATGCTGCCTGCTCTTAC	TGGGAAGTGGGTGCAGTTATTGTC
*Nrf2*	TAAAGCACAGCCAGCACATTCTCC	TGATGACCAGGACTCACGGGAAC
*Nqo1*	GCTGGTTTGAGAGAGTGCTCGTAG	CCCGTGGACACCCTGAAGAGAG
*Cat*	GGAGGCGGGAACCCAATAGGAG	TCAAAGTGTGCCATCTCGTCAGTG
*Nlrp3*	GAGCTGGACCTCAGTGACAATGC	ACCAATGCGAGATCCTGACAACAC
*Casp1*	CATCCTGTCAGGGGCTCACTTTTC	CTATCAGCAGTGGGCATCTGTAGC
*Asc*	GAAGTGGACGGAGTGCTGGATG	CTTGTCTTGGCTGGTGGTCTCTG
*Txnip*	CCCAGATACCCCAGAAGCTCCTC	TGAGAGTCGTCCACATCGTCCAG
*Il1β*	CAAGAGCTTCAGGCAGGCAGTATC	AGGTCCACGGGAAAGACACAGG
*Il18*	GGCTGCCATGTCAGAAGACTCTTG	AGTGAAGTCGGCCAAAGTTGTCTG
*Sod2*	AGCCGTGTCTGTGGGAGTCC	AGAGCAGGCAGCAATCTGTAAGC
*Il17*	GCCAAGGACTTCCTCCAGAATGTG	TGGAACGGTTGAGGTAGTCTGAGG
*Ifnγ*	AGGAACTGGCAAAAGGATGGTGAC	GTTGTTGCTGATGGCCTGATTGTC

### Western Blot Assay

The spinal cords tissues were lysed in ice-cold RIPA lysis buffer (Beyotime Biotechnology, Shanghai, China). The protein concentration was determined using a BCA reagent kit (Beyotime Biotechnology, Shanghai, China). Total protein (30 μg) was separated by 10% sodium dodecyl sulfate-polyacrylamide gel electrophoresis, and transferred onto PVDF membranes (Millipore, Billerica, MA, USA). The membranes were blocked in tris-buffered saline with 5% non-fat milk and 0.5% bovine serum albumin for 1 h at room temperature and then incubated overnight at 4°C with primary antibodies (1:1,000). After washing, the membranes were incubated with secondary antibodies (1:5,000) for 1 h at 37°C. Blots were visualized with the Chemiluminescent HRP substrate (Millipore) and quantified with the Quantity 5.2 software System (Bio-Rad).

### Statistical Analyses

All data are expressed as mean ± SD. Statistical analysis was performed using GraphPad Prism 7.0 software (GraphPad, San Diego, CA, USA) with one-way ANOVA, followed by *post-hoc* multiple comparisons with the Tukey’s test. Statistical significance was considered as P < 0.05.

## Results

### Bixin Attenuates the Symptoms of EAE Mice

**Figure 1:**
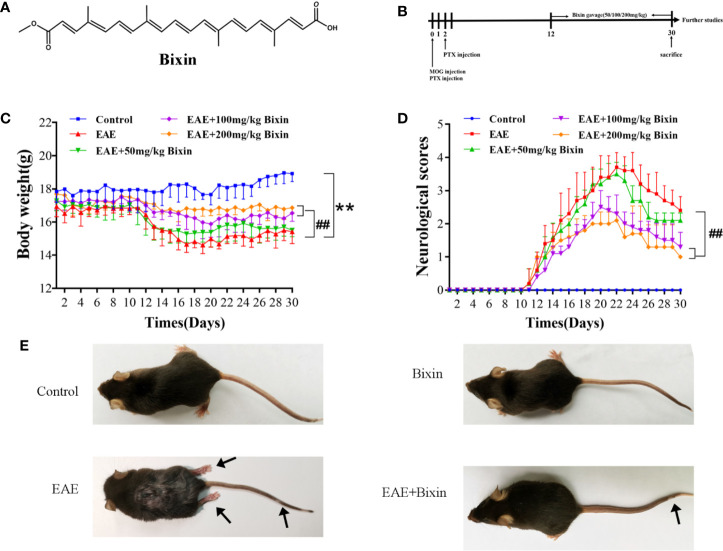
Bixin attenuates the symptoms of EAE mice **(A)**. Chemical structure of bixin **(B)**. Study procedure **(C)**. Body weight and **(D)** clinical behavior scores of the EAE mice **(E)**. Representative images show behavioral symptoms of EAE mice in each group. Black arrows present limp tail or hind limb paralysis. Data shown in graphs represents the means ± SD (n = 5). **P < 0.01, ***vs.*** control group; ^##^P < 0.01, ***vs.*** EAE group.

To evaluate the effects of bixin on the symptoms of EAE mice (**Figures 1A, B**), daily weight changes and clinical behavioral scores were recorded. The results showed that bixin treatment significantly delayed the detrimental effects of EAE on body weight (**Figure 1C**). Furthermore, bixin treatment dose-dependently reduced the clinical symptoms in the EAE mice (**Figure 1D**). At the same time, we found that EAE mice showed severe clinical signs with flaccid tail and complete paralysis of the hindlimbs; while, bixin-treated EAE mice only showed tail paralysis (**Figure 1E**). These results indicated that the appropriate dose of bixin to attenuate the symptoms of EAE was 100 mg/kg, which was used for subsequent experiments.

### Bixin Reduces the Inflammation and Demyelination in EAE Mice

**Figure 2:**
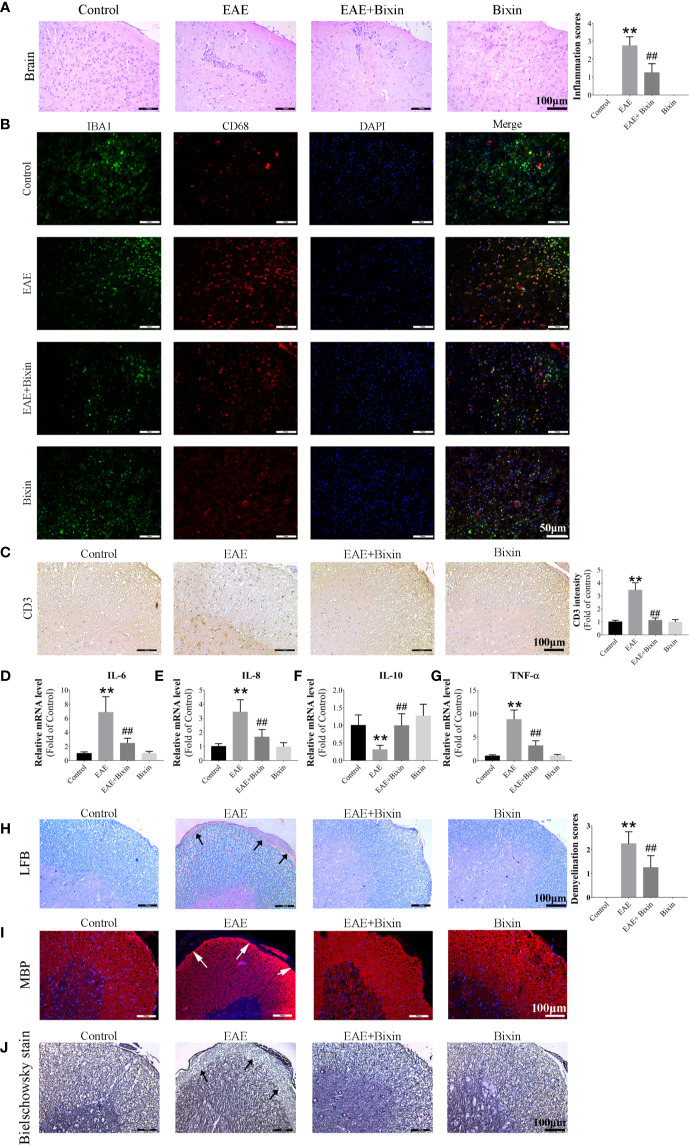
Bixin reduces the inflammation and demyelination in EAE mice **(A)**. H&E staining showing inflammatory cell infiltration in the brain, as well as the inflammation scores. Scale bars: 100 μm **(B)**. Representative IF images of IBA1 and CD68 in brain sections showing the activated microglia. Scale bars: 50 μm **(C)**. Representative IHC images of CD3+ cells in the spinal cord. Scale bars: 100 μm **(D–G)**. mRNA expression of the inflammatory cytokines IL-6, IL-8, IL-10, and TNF-α were quantitated by qRT-PCR **(H)**. LFB staining showing demyelination in the spinal cord, as well as the demyelination scores. Scale bars: 100 μm **(I)**. IF staining of MBP in the spinal cords. Scale bars: 100 μm **(J)**. Bielschowsky staining showing the degree of axon degeneration. Scale bars: 100 μm. Data shown in graphs represents the means ± SD (n = 5). **P < 0.01, ***vs.*** control group; ^##^P < 0.01, ***vs.*** EAE group.

Previous studies have indicated that inflammation and demyelination are the primary features in EAE mice (5). Our results showed that EAE significantly increased the number of inflammatory cells and inflammation scores in the brain, however, bixin treatment noticeably reduced both of these effects (**Figure 2A**). Since the multiple inflammatory cells in the CNS which is important in MS study. Microglia are the main mediators of neuronal inflammation (25) and the lysosomal protein CD68 is highly expressed on the surface of activated microglia (5), we evaluated the co-localized expression of IBA1 and CD68 by IF. In line with the above results, although EAE significantly upregulated the levels of IBA1 and CD68, bixin treatment remarkably suppressed this phenotype (**Figure 2B**). And we detected the expression of CD3+ cells by IHC, the results showed that EAE significantly increased the expression of CD3+ cells, bixin significantly decreased the expression of CD3+ cells (**Figure 2C**).

Given that inflammatory cytokines play key roles in inflammation, we also evaluated the levels of several cytokines by qRT-PCR in the spinal cords and found that the mRNA levels of pro-inflammatory cytokinesr TNF-α, IL-6, and IL-8, were markedly increased in the spinal cords of EAE mice, while that of the anti-inflammatory cytokine IL-10 was decreased. Meanwhile, bixin treatment reduced the mRNA levels of TNF-α, IL-6, and IL-8, and increased that of IL-10 (**Figures 2D–G**).

Furthermore, LFB staining revealed that bixin treatment reduced the level of spinal cord demyelination in EAE mice (**Figure 2H**). Similarly, IF staining showed that bixin-treated EAE mice had significantly increased expression of MBP, a structural protein of myelin (**Figure 2I**). Meanwhile, we determined the degree of axon degeneration in the CNS by Bielschowsky staining. The results showed that bixin treatment significantly decreased the degree of axon degeneration (**Figure 2J**).

### Bixin Reduces the Percentages of Th1 and Th17 Cell Subsets

**Figure 3:**
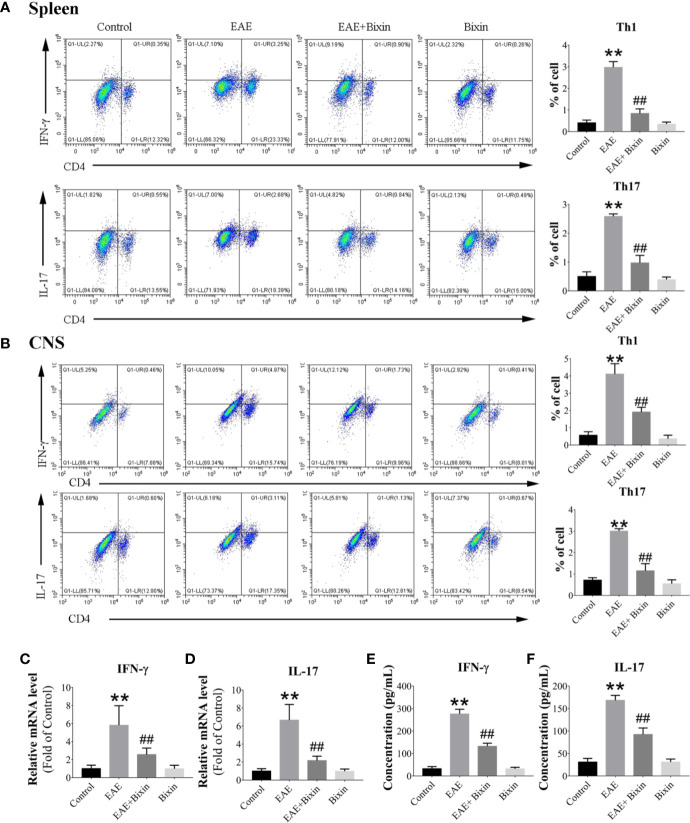
Bixin reduces the proportion of Th1 and Th17 cell subsets. Subsets of Th1 and Th17 cells in CD4+ gate were analyzed by intracellular staining for IFN-γ and IL-17, respectively, in MNCs from the spleen **(A)** and CNS **(B)**. **(C, D)**. mRNA expression of the inflammatory cytokines IFN-γ and IL-17 were quantitated by qRT-PCR **(E–F)**. IFN-γ and IL-17 levels in serum were measured by ELISA. Data shown in graphs represents the means ± SD (n = 5). **P < 0.01, ***vs.*** control group; ^##^P < 0.01, ***vs.*** EAE group.

Previous studies have demonstrated that T helper (Th) 1 and Th17 cells are responsible for the inflammatory demyelination in both MS and EAE. Th 1 cells primarily produce pro-inflammatory cytokines IFN-γ, while Th 17 cells secrete IL-17 (24, 26). Therefore, we evaluated the proportions of Th1 and Th17 cell subsets in the spleen and CNS by flow cytometry. Both Th1 and Th17 cell subsets in the spleen and CNS of EAE mice were significantly increased compared to the control group, while bixin treatment remarkably reduced the proportion of both cell subsets (**Figures 3A, B**). Meanwhile, we quantified the IFN-γ and IL-17 levels by qRT-PCR and ELISA, and found that EAE significantly upregulated the levels of IFN-γ and IL-17, while bixin treatment remarkably reduced the expression of both (**Figures 3C–F**).

### Bixin Suppresses Activation of the TXNIP/NLRP3 Inflammasome in EAE Mice

**Figure 4:**
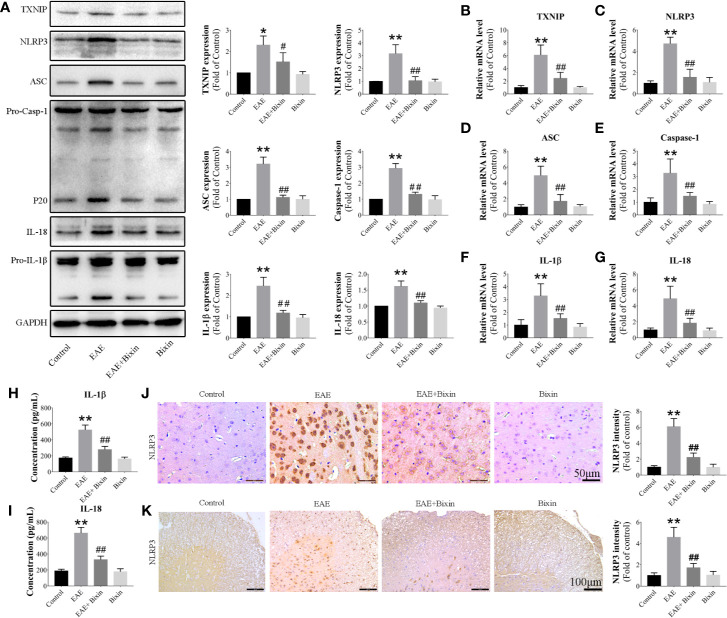
Bixin suppresses the activation of the TXNIP/NLRP3 inflammasome in EAE mice **(A)**. The protein levels of TXNIP/NLRP3 inflammasome components were assessed by western blotting **(B–G)**. mRNA expression of TXNIP/NLRP3 inflammasome components were quantitated by qRT-PCR **(H, I)**. IL-1β and IL-18 levels in serum were measured by ELISA. Representative IHC images of NLRP3 in brain sections **(J)** (Scale bars: 50 μm) and spinal cord sections **(K)** (Scale bars: 100 μm), and the quantitative results are shown. Data shown in graphs represents the means ± SD (n = 5). *P < 0.05, **P < 0.01, ***vs.*** control group; ^#^P < 0.05, ^##^P < 0.01, ***vs.*** EAE group.

To clarify whether bixin suppresses the inflammatory response in EAE mice by inhibiting the activation of the TXNIP/NLRP3 inflammasome, we evaluated the individual protein component levels for this complex in the spinal cords by western blotting. The results showed that TXNIP, NLRP3, ASC, caspase-1, IL-1β, and IL-18 levels were increased in EAE mice, and bixin treatment suppressed this effect (**Figure 4A**). The same results were observed for the mRNA levels of these components by qRT-PCR (**Figures 4B–G**). Furthermore, IL-1β and IL-18 levels in serum were increased in EAE mice, and bixin treatment reduced their upregulation (**Figures 4H, I**). These findings were corroborated by IHC; although NLRP3 were highly expressed in the CNS of EAE mice, bixin treatment remarkably inhibited their expression (**Figures 4J, K**).

### Bixin Inhibits Oxidative Stress in EAE Mice

**Figure 5:**
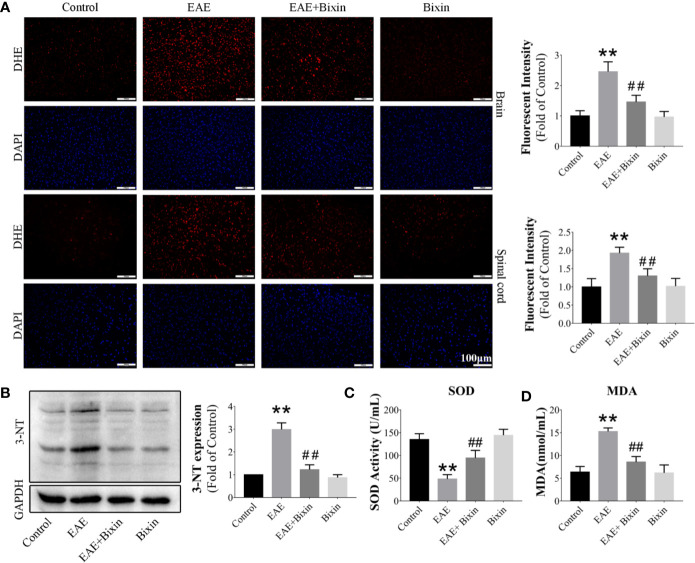
Bixin alleviates oxidative stress in EAE mice **(A)**. The level of reactive oxygen species (ROS) in the brain and spinal cord tissues was assessed by DHE staining, and the quantitative results are shown. Scale bars: 100 μm **(B)**. The protein level of 3-NT in the CNS tissues was assessed by western blotting, and the quantitative results are shown **(C)**. The activities of SOD and **(D)** the levels of MDA in the serum. Data shown in graphs represents the means ± SD (n = 5). **P < 0.01, ***vs.*** control group; ^##^P < 0.01, ***vs.*** EAE group.

To elucidate whether the anti-inflammatory effect of bixin occurred through an anti-oxidative effect, we evaluated the ROS levels in the brain and spinal cord by DHE staining. The results showed that ROS levels were significantly increased in EAE mice, and bixin treatment alleviated this oxidative stress (**Figure 5A**). Additionally, western blotting results showed that the level of the oxidative damage marker 3-NT in EAE mice was reduced by bixin treatment (**Figure 5B**). Furthermore, bixin treatment reduced the level of MDA, while increasing the expression of SOD in EAE mice (**Figures 5C, D**).

### Bixin Activates the NRF2 Signaling in EAE Mice

**Figure 6:**
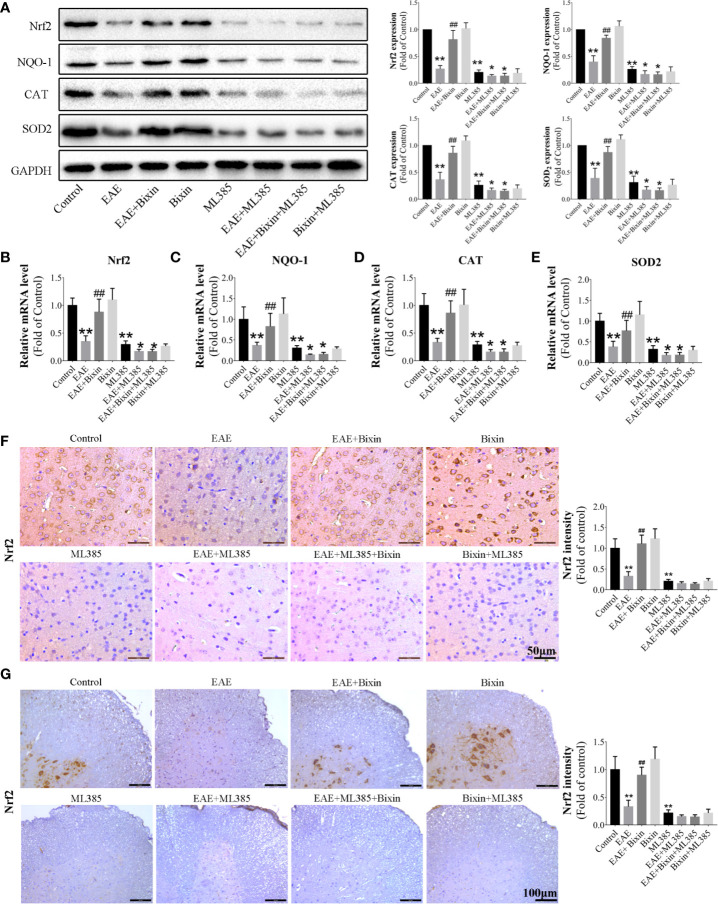
Bixin activates the NRF2 signaling in EAE mice **(A)**. The protein levels of NRF2 and downstream genes Catalase, NQO-1, and SOD2 in the brain tissues were assessed by western blotting, and the quantitative results are shown **(B–E)**. mRNA expression of NRF2, Catalase, NQO-1, and SOD2 in the CNS tissues were quantitated by qRT-PCR. IHC of NRF2 in brain sections **(F)** (Scale bars: 50 μm) and spinal cord sections **(G)** (Scale bars: 100 μm). Data shown in graphs represents the means ± SD (n = 5). *P < 0.05, **P < 0.01, ***vs.*** control group or ML385 group; ^##^ P < 0.01, ***vs.*** EAE group or EAE + ML385 group.

Since NRF2 plays a critical role in oxidative stress response (27), we next assessed the effect of bixin on NRF2. To this end, we evaluated the levels of NRF2 signaling components by western blotting and found that although the levels of NRF2, and its downstream proteins, catalase, NQO-1, and SOD2 were reduced in EAE mice, bixin treatment significantly upregulated their expression (**Figure 6A**). Similarly, the mRNA levels had the same trends in expression (**Figures 6B–E**). Furthermore, IHC indicated that NRF2 expression was significantly reduced in EAE mice, and bixin treatment reversed this phenotype (**Figures 6F, G**).

**Figure 7:**
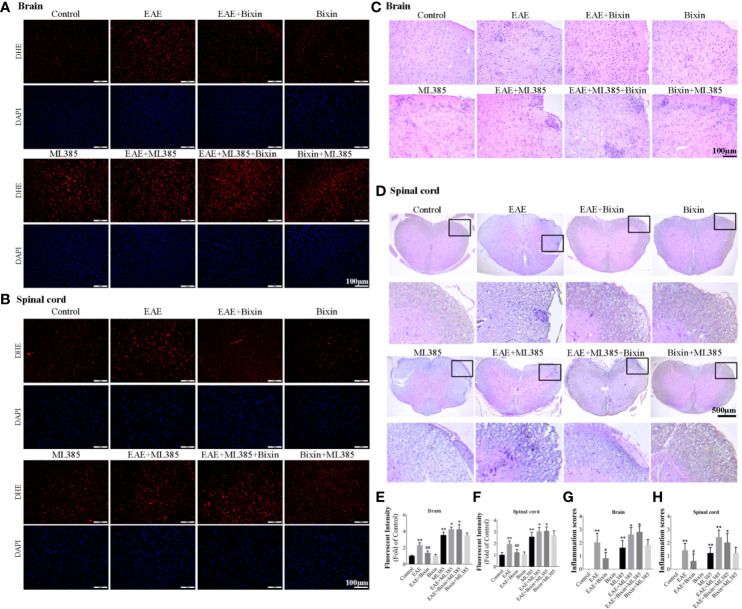
Bixin suppressed the production of ROS and inflammation in EAE mice by activating NRF2 signaling. The level of reactive oxygen species (ROS) in the brain tissues **(A)** and spinal cord tissues **(B)** was assessed by DHE staining, as well as the quantitative results **(E, F)**. Scale bars: 100 μm. H&E staining showing inflammatory cell infiltration in the brain **(C)** (scale bars: 100 μm) and the spinal cord **(D)** (scale bars: 500 μm), as well as the inflammation scores **(G, H)**. Data shown in graphs represents the means ± SD (n = 5). *P < 0.05, **P < 0.01, *vs.* control group or ML385 group; ^#^ P < 0.05, ^##^ P < 0.01, *vs.* EAE group or EAE + ML385 group.

To confirm the pivotal role of NRF2 in the bioactivity of bixin, the NRF2 inhibitor ML385 was intraperitoneally administered 1 h before intragastric administration of bixin, which significantly decreased the expression of NRF2 and its downstream genes, and increased the levels of ROS (**Figures 7A, B, E, F**) and inflammation (**Figures 7C, D, G, H**). However, bixin could not reverse this phenotype in ML385-treated EAE mice (**Figure 7**).

## Discussion

MS is a characteristic autoimmune inflammatory demyelinating disease of the CNS that significantly impacts the quality of life of those affected (28). While numerous drugs are available for MS treatment, their efficacy remains unsatisfactory. Hence, numerous researchers have sought to better understand the pathophysiology of this disease to inform the design of improved therapeutics. To this end, Dang C et al. (29) reported that upregulating PGC-1α significantly improves the survival ability of neurons in EAE *via* inhibiting oxidative stress. Additionally, ghrelin decreases neuroinflammation and demyelination by inhibiting the NLRP3 inflammasome activity (5). In this study, we similarly demonstrated that NLRP3 inflammasome activity and oxidative stress were the key factors in EAE development.

Bixin is a natural carotenoid with multiple bioactivities. Previous studies have shown that bixin has anti-inflammation, anti-tumor, and anti-oxidative effects (30–34). Xu Z et al. (19) have found that bixin attenuates cardiac injury by inhibiting inflammation and oxidative stress in a high-fat-diet mouse model. In this study, we found that bixin, a natural carotenoid, suppressed inflammatory cell infiltration and the levels of TNF-α, IL-6, IL-8, IL-17, and IFN-γ, while upregulating the expression of IL-10. Microglia are resident immune effector cells in the brain that are easily activated resulting in chronic neuroinflammatory reaction in the brain. What’s more, these cells are closely associated with the development of neurodegenerative diseases, such as Alzheimer’s disease (31, 35). In our study, we found that EAE upregulated the expression of IBA1 and CD68, markers of activated microglia; however, bixin was found to reduce microglia activation in the brain. And bixin significantly decreased the expression of CD3+ cells. These results indicated that bixin improved the symptoms in EAE mice and reduced demyelination by inhibiting inflammatory cell infiltration and the production of inflammatory cytokines.

Autoreactive pathogenic T lymphocytes have also been described as being closely related to the development and progression of MS and EAE (26). Specifically, activated CD4+ T cell subsets produce a large number of inflammatory mediators, such as ROS, which induces subsequent oxidative stress and inflammatory demyelination in EAE. Recent studies have found that CD4+ T subsets play key roles in MS and EAE pathogenesis, including Th1 cells, which primarily secrete pro-inflammatory cytokines IFN-γ, and Th17 cells, which produce IL-17 (23, 24, 26, 36). In our study, we found that EAE increased the proportion of Th1 and Th17 cell subsets in the spleen and CNS, resulting in corresponding increased levels of IL-17 and IFN-γ, while bixin treatment markedly inhibited these effects. These results indicated that bixin reduced neuroinflammation in EAE *via* an immune regulatory mechanism.

The TXNIP/NLRP3 inflammasome plays a key role in the pathogenesis of various diseases (32, 34, 37, 38). Chen W. et al. (9) indicated that minocycline improves diabetic retinopathy by inhibiting the TXNIP/NLRP3 inflammasome pathway, and vitamin D3 attenuates diabetic retinopathy by inhibiting high-glucose-induced ROS/TXNIP/NLRP3 inflammasome pathway (39). Emerging evidence suggests that the NLRP3 inflammasome, composed of NLRP3, ASC, and caspase-1, plays a critical role in the pathogenesis of MS and EAE (40, 41). And recent researches have reported that NLRP3 inflammasome inhibitor JC-171 and OLT1177 were a potential therapeutic agent for MS (34, 42). Liu F et al. (5) found that Ghrelin attenuated MS by suppressing the activation of NLRP3 inflammasome. In the present study, we also found that the TXNIP/NLRP3 inflammasome components were upregulated in EAE mice, and bixin significantly downregulated the expression of TXNIP and NLRP3 at both the mRNA and protein levels. These results indicated that bixin reduced neuroinflammation in EAE by suppressing the activation of the TXNIP/NLRP3 inflammasome.

Recent studies have reported that oxidative stress is one of the main causes of CNS dysfunction in MS (27). ROS are also the main mediators of oxidative stress and initiators of the TXNIP/NLRP3 inflammasome (43–45). In our study, we found that bixin attenuated ROS accumulation, downregulated the expression of oxidative damage marker 3-NT and the levels of MDA in EAE mice, and increased the expression of SOD. These results indicated that bixin suppressed the activation of the TXNIP/NLRP3 inflammasome through the attenuation of oxidative stress.

NRF2, an important redox sensor, binds to antioxidant response elements and counteracts the production of ROS by activating the expression of many antioxidant genes (46, 47). Meanwhile, bixin has been reported to alleviate photodamage and hair graying by activating NRF2 signaling (48); and to promote tissue repair and improve pulmonary injury induced by particle exposure in a NRF2-dependent manner (20, 49). In our study, the results showed that bixin markedly upregulated NRF2 and its downstream antioxidant target genes in EAE mice. These results indicated that bixin suppressed the production of ROS by activating NRF2 signaling. Furthermore, we found that the protective role of bixin was dependent on NRF2 activation, as the NRF2 inhibitor ML385 caused near complete abrogation of the effects elicited by bixin on ROS attenuation. Therefore, these findings indicated that bixin prevented neuroinflammation and demyelination in EAE mice primarily by scavenging ROS through activation of the NRF2 signaling pathway.

## Conclusions

Bixin inhibits the TXNIP/NLRP3 inflammasome and activates the NRF2 signaling pathway in EAE mice. While bixin may be a possible therapeutic strategy for MS, further mechanistic studies *in vitro* are necessary.

## Data Availability Statement

The datasets presented in this study can be found in online repositories. The names of the repository/repositories and accession number(s) can be found in the article/**Supplementary Material**.

## Ethics Statement

The animal study was reviewed and approved by the Medical Ethics Committee of Chengdu Medical College.

## Author Contributions

YY and D-MW researched data. YY and D-MW wrote, reviewed, and edited the manuscript. JL, S-HD, TL, TZ, MH, and Y-YZ provided research material and techniques. YX directed the project, and wrote, reviewed, and edited the manuscript. All authors contributed to the article and approved the submitted version.

## Funding

This study was funded by the National Natural Science Foundation of China (81972977 and 81802955), Foundation of Sichuan Science and Technology Agency (2018JY0648 and 2019YJ0589), Foundation of The First Affiliated Hospital of Chengdu Medical College (CYFY2017ZD03 and CYFY2018ZD02), Foundation of Collaborative Innovation Center of Sichuan for Elderly Care and Health, Chengdu Medical College (19Z01), and the Foundation of Chengdu Medical College (CYFY2019ZD06).

## Conflict of Interest

The authors declare that the research was conducted in the absence of any commercial or financial relationships that could be construed as a potential conflict of interest.
