# Supplementary figures and images for: Bixin Attenuates Experimental Autoimmune Encephalomyelitis by Suppressing TXNIP/NLRP3 Inflammasome Activity and Activating NRF2 Signaling
Source: Front Immunol. 2020 Dec 9;11:593368. doi: 10.3389/fimmu.2020.593368 (PMC7756000; doi:10.3389/fimmu.2020.593368)

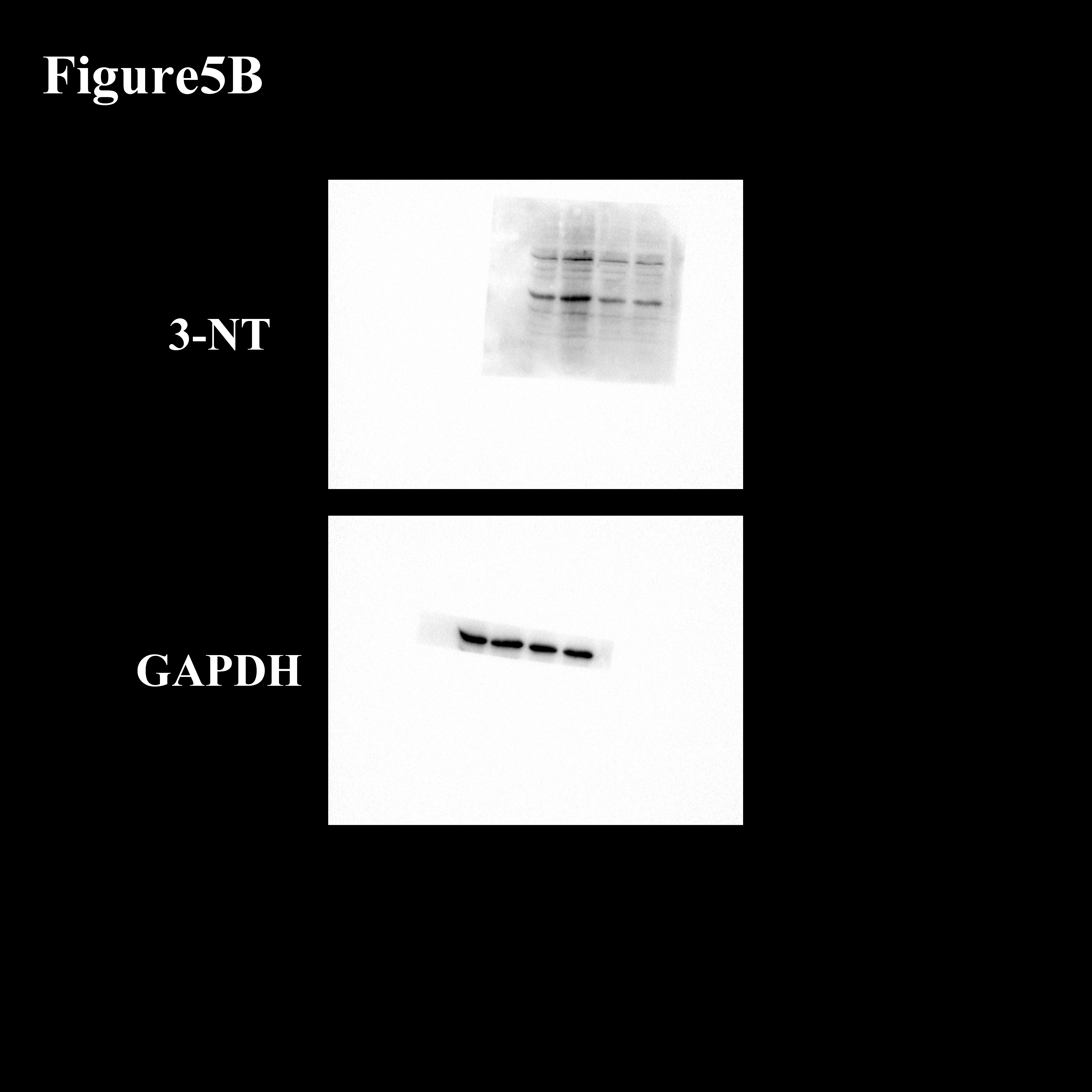

Supplement: Supplementary file 1 [file DataSheet_1.zip › original western blot/Figure5B.tif]

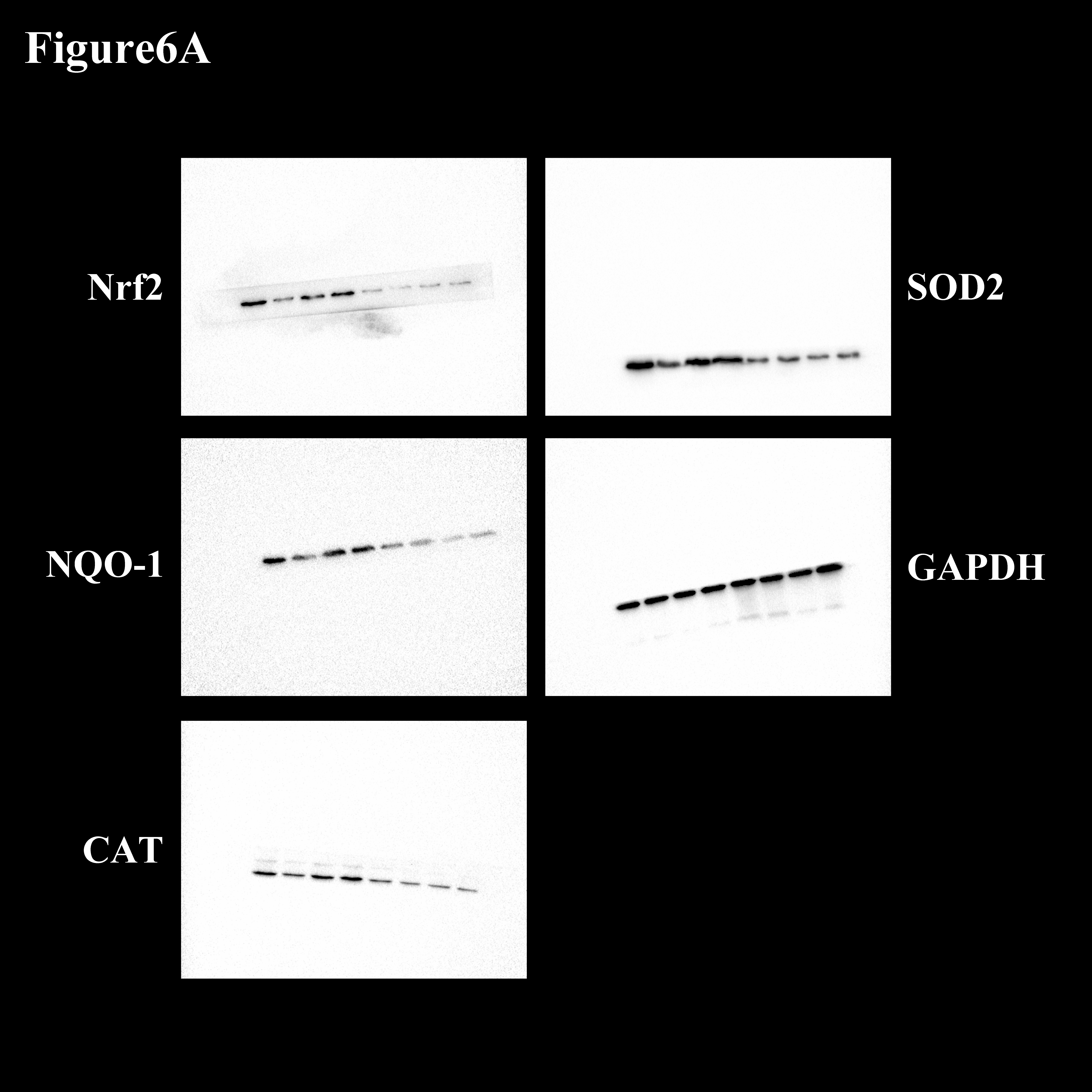

Supplement: Supplementary file 1 [file DataSheet_1.zip › original western blot/Figure6A.tif]
